# Patients with myelodysplastic/myeloproliferative neoplasms have higher rates of relapse following allogeneic hematopoietic cell transplantation compared to those with MDS: A Brazilian SBTMO/CIBMTR registry analysis

**DOI:** 10.1038/s41409-026-02877-2

**Published:** 2026-04-24

**Authors:** Fernando Barroso Duarte, Yhasmine Delles Oliveira Garcia, Vanderson Rocha, Vaneuza Araújo Moreira Funke, Maria Claudia Rodrigues Moreira, Alessandra Aparecida Paz, Jayr Schmidt Filho, Claudia Caceres Astigarraga, Roberto Luiz da Silva, Vinícius Campos de Molla, Alexandre Silvério, João Victor Piccolo Feliciano, George Maurício Navarro Barros, Vergílio Antônio Rensi Colturato, Samir Kanaan Nabhan, João Samuel de Holanda Farias, Ana Carolina Arrais Maia, Ângelo Atalla, Ricardo Chiattone, Maria Cristina Martins de Almeida Macedo, Milton Alexandre Ferreira Aranha, Yana Augusta Novis Zogbi, Décio Lener, Rodolfo Daniel de Almeida Soares, Phillip Scheinberg, Rodolfo Froes Calixto, Gustavo Machado Teixeira, Marcos Paulo Colella, Celso Arrais Rodrigues, Anderson João Simione, Cinthya Corrêa da Silva, Nelson Hamerschlak

**Affiliations:** 1 Federal University of Ceará Walter Cantídio Hospital, Fortaleza, Brasil; 2 Centro de Transplante de Medula Óssea do Hospital das Clínicas da Faculdade de Medicina da USP - CTMO-HCFMUSP, São Paulo, Brasil; 3 Hospital Nossa Senhora das Graças, Curitiba, Brasil; 4 Complexo Hospitalar de Niterói, Niterói, Brasil; 5 Centro Nacional de Transplante de Medula Óssea (CEMO), Instituto Nacional de Câncer, Rio de Janeiro, Brasil; 6 Hospital de Clínicas de Porto Alegre, Porto Alegre, Brasil; 7 A.C. Camargo Cancer Center, Sao Paulo, Brasil; 8 Associação Hospitalar Moinhos de Vento, Porto Alegre, Brasil; 9 Bio Sana’s Serviços Médicos, São Paulo, Brasil; 10 Centro de Pesquisa Clínica Hospital 9 De Julho, São Paulo, Brasil; 11 Centro de Pesquisas Oncológicas Dr. Alfredo Daura Jorge (CEPON), Florianópolis, Brasil; 12 Fundação Faculdade Regional de Medicina de São José do Rio Preto (FUNFARME), São José do Rio Preto, Brasil; 13 Fundação Pio XII - Hospital de Câncer de Barretos, Barretos, Brasil; 14 Hospital Amaral Carvalho, Jaú, Brasil; 15 Hospital de Clínicas – UFPR, Curitiba, Brasil; 16 Hospital Erasto Gaertner, Curitiba, Brasil; 17 Hospital Leforte Liberdade, São Paulo, Brasil; 18 Hospital Monte Sinai, Juiz de Fora, Brasil; 19 Hospital Samaritano, São Paulo, Brasil; 20 Hospital São Camilo – Mooca, São Paulo, Brasil; 21 Hospital São Camilo – Pompéia, São Paulo, Brasil; 22 Hospital Sírio Libanês, São Paulo, Brasil; 23 Natal Hospital Center, Natal, Brasil; 24 Real e Benemérita Sociedade de Beneficiência Portuguesa de São Paulo, São Paulo, Brasil; 25 Real Hospital Português, Recife, Brasil; 26 UFMG Hospital das Clínicas Servico de Transplante de Medula Óssea, Belo Horizonte, Brasil; 27 UNICAMP – HEMOCENTRO, Campinas, Brasil; 28 Universidade Federal de São Paulo - Hospital São Paulo, São Paulo, Brasil; 29 Albert Einstein Hospital, São Paulo, Brasil

**Keywords:** Myelodysplastic syndrome, Myeloproliferative disease

**Correspondence**

The Myelodysplastic/Myeloproliferative neoplasm (MDS/MPN) overlap syndromes are a heterogeneous group of rare hematologic malignancies characterized by features of both MDS such as morphologic dysplasia and cytopenias and MPNs including elevated blood counts and often splenomegaly [1]. These syndromes frequently harbor recurrent mutations, underscoring their interrelated biological nature includes genes involved in epigenetic regulation, splicing, signaling, and gene transcription[1]. Mutational signatures can help differentiate among the different MDS/MPN subtypes. In chronic myelomonocytic leukemia (CMML), mutations in *TET2, SRSF2*, and/or *ASXL1* are seen in the vast majority of cases, with the combination of *TET2* and *SRSF2* mutations or bi-allelic *TET2* mutations being characteristic [1, 2]. In contrast to MDS, *TP53* alterations are exceedingly rare in MDS/MPN [1]_._ The prognosis for patients varies widely based on the specific subtype, molecular landscape, and individual patient factors [2].

The allogeneic hematopoietic cell transplantation (HCT) remains the only curative therapy, but is only available in a fraction of patients despite inherent risks to advanced age and comorbidities [1, 3]. The rarity and heterogeneity of these disorders that no standardized management approach for these conditions, particularly concerning the indications for and management of HCT, including optimal patient selection, pre-transplant treatment strategies and appropriate timing of transplantation. Current recommendations emphasize the early identification of potential transplant candidates, especially in atypical chronic myeloid leukemia (aCML), MDS/MPN unclassifiable (MDS/MPN-U) and in cases with specific molecular patterns present in some of these diseases [4].

This study included all pediatric and adult patients diagnosed with MDS who underwent their first HCT in Brazil, from any donor type, between 2012 to 2024, as reported for 33 centers to the Brazilian Registry of Hematopoietic Cell Transplantation and Cell Therapy Society/Center for International Blood and Marrow Transplant Research (RBTCH-TC/CIBMTR) using the electronic FormsNet3 platform. Patients who did not undergo a first transplant, had missing follow-up, unavailable post-transplant relapse information, or missing relapse dates were considered exclusion criteria. In total, data from 570 patients were available for analysis. For outcome analysis, patients were stratified according to the WHO 2016 classification, chosen given the study’s retrospective design in the MDS/MPN group included patients, such as CMML, aCML, MDS/MPN with ring sideroblasts and thrombocytosis (MDS/MPN-RS-T), and (MDS/MPN-U), whereas the MDS group comprised all remaining other types of MDS, which are detailed in the supplementary material.

Survival was estimated using the Kaplan-Meier method and results were compared with log-rank test (*p* < 0.05). Univariable and multivariable analysis was performed by proportional hazards regression, using R Statistical Software (V.4.2.1). Ethical approval was obtained in 2019 (Conep CAAE: 65575317.5.1001.0071). Methods are detailed in supplemental material.

Of 570 included patients, 476 (83.5%) had other MDS classifications. and 94 (16.5%) had MDS/MPN, which most frequent were MDS/MPN-U (60.6%), followed by CMML (29.8%), MDS/MPN with *SF3B1* and thrombocytosis (3.2%), and aCML (2.1%). Patients’ characteristics are resumed in Supplemental Table 1 (Table S1). When comparing baseline characteristics between groups, MDS/MPN patients were significantly older (*p* < 0.001) and more frequently had KPS ≤ 80 (*p* = 0.004) compared with MDS patients (Table S1).

At 25 months post-transplant, the overall survival (OS) was significantly lower in the MDS/MPN group compared with the MDS group (41.9% vs. 59.7%; *p* = 0.03; Fig. 1A). Relapse Free Survival (RFS) was also inferior in MDS/MPN patients (39.2% vs. 56.6%, *p* = 0.003; Fig. 1B). At 25 months, Non-Relapse Mortality (NRM) was 29.0% for MDS and 35.2% for MDS/MPN, with no statistically significant between-group difference (*p* = 0.331) (Fig. 1C).

**Fig. 1:**
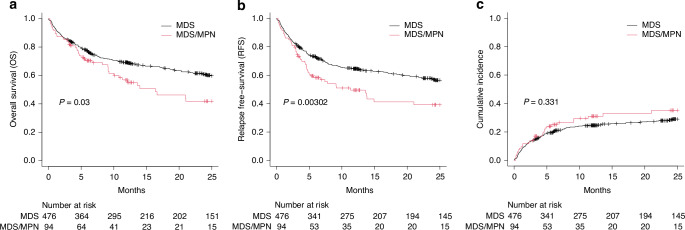
Overall Survival (OS), Relapse-Free Survival (RFS) and Non-relapse mortality (NRM) after first HCT in patients with MDS (*n* = 476) versus MDS/MPN (*n* = 94). **a** The survival estimates at 25 months were 59.7% (95% CI, 54.7–64.4) for patients with MDS (black line), and 41.9% (95% CI, 29.3%-53.9%) for patients with MDS/MPN (red line). **b** The survival estimates at 25 months were 56.5% (95% CI, 51.5–61.2) for patients with MDS (black line), and 39.2% (95% CI, 27.5%–50.7%) for patients with MDS/MPN (red line). **c** The cumulative incidence of relapse estimates at 25 months were 29% for patients with MDS (black line), 35.2% for patients with MDS/MPN (red line).

In univariate analysis, the MDS/MPN subtype was associated with inferior OS (HR = 1.44, 95% CI 1.03–2.00, *p* = 0.031) and RFS (HR = 1.58, 95% CI 1.16–2.14, *p* = 0.003). NRM, was no statistically significant difference (HR = 1.21, 95% CI 0.81–1.79, *p* = 0.353). In multivariable analysis, MDS/MPN remained an independent risk factor for inferior RFS (HR = 1.35, 95% CI 1.03–1.88; *p* = 0.0079) (Table S2).

MDS/MPN represent a rare group of hematologic disorders [2]. Although their relative incidence is globally low [2, 4–6], in the present study only 16.5% of all patients had MDS/MPN. In an analysis global of MDS/MPN using data from the Global Burden of Disease 2021, a worldwide increase in MDS/MPN cases, mainly attributed to population growth and aging [5]. In the same study, the greatest burden of MDS/MPN was found among men and older populations [5], which is consistent with the findings of the present study, in which most patients were male and had a median age of 59 years, higher than that of the MDS group (50 years), corroborating a similar study that reported a median age of 61 years in patients with MDS/MPN [3].

Although CMML is the most common MDS/MPN subtype [1], in the present study we observed a higher frequency of cases classified as MDS/MPN-U. Being a diagnosis of exclusion, MDS/MPN-U encompasses cases with different characteristics that do not meet the criteria for other subtypes and are therefore included in this heterogeneous category [6]. In addition, within MDS/MPN entities, conventional cytogenetics can identify clonality/aberrations in 15% to 35% of cases, while NGS panels show recurrent myeloid gene mutations in approximately 90% of cases [4]_._ However, access to molecular test tools still represents a challenge in Brazil [7].

In this cohort, compared with patients with MDS, MDS/MPN recipients were older and had lower KPS, reflecting a more unfavorable clinical profile at the time of transplant. The prognosis for patients with MDS/MPNs varies widely based on the specific subtype, molecular landscape, and individual patient factors [4].

In the present study, the OS for patients with MDS/MPN was 41%, and RFS was 39.2%, both significantly lower than those observed in other MDS types. The cumulative incidence NRM was 35.2% and did not show a statistically significant difference between the MDS and MDS/MPN groups (*p* = 0.331). However, with a Hazard Ratio of 1.21 (95% CI: 0.81–1.79; *p* = 0.353), is suggested a trend towards a higher risk of NRM in patients with MDS/MPN. This lack of significance is attributed to unbalanced and relatively small sample size of the MDS/MPN group and as well as the low number of NRM events. These outcomes are comparable, to those reported in a retrospective single-center study including 75 MDS/MPN patients, in which OS at 24 months was 51.6%, RFS was 24.8%, and NRM was 37.9% among patients receiving either ATG or post-transplant cyclophosphamide (PTCy) for GVHD prophylaxis [3]. In that cohort, poor KPS was associated with inferior OS, higher NRM, and worse graft-versus-host/relapse-free survival in univariate analysis [3].

In univariate analysis, MDS/MPN was associated with inferior OS and RFS compared with other MDS types. In multivariable analysis, MDS/MPN remained an independent risk factor for worse RFS, underscoring its negative prognostic impact. These findings align with previous studies reporting poorer outcomes for patients with MDS/MPN-U (*n* = 85) compared with other MDS types (*n* = 2241), and primary myelofibrosis (*n* = 600) (*p* < 0.001) [8]. In addition, age, Hb, thrombocytosis, IPSS-R, and mutation with *SETBP1* and *SRSF2* have been proposed as predictors of survival in other studies with patients MDS/MPN [8, 9].

Limitations of the study include its retrospective nature, heterogeneous patient characteristics, limited sample size, and variability in diagnostic accuracy and access to molecular testing across centers. In conclusion, recipients of HCT for MDS/MPN were older, had poorer performance status, and experienced inferior OS and RFS compared with patients transplanted for other MDS types. These findings confirm the adverse prognosis of MDS/MPN and underscore the need for further studies aimed at optimizing conditioning strategies, donor selection, and post-transplant management to improve curative outcomes for this challenging patient population.

## Supplementary information

SUPPLEMENTAL MATERIAL
