# Supplementary material for: Patients with myelodysplastic/myeloproliferative neoplasms have higher rates of relapse following allogeneic hematopoietic cell transplantation compared to those with MDS: A Brazilian SBTMO/CIBMTR registry analysis
Source: Bone Marrow Transplant. 2026 Apr 24;61(8):993–5. doi: 10.1038/s41409-026-02877-2 (PMC13282737; doi:10.1038/s41409-026-02877-2)
Supplement: Supplementary file 1 [file 41409_2026_2877_MOESM1_ESM.docx]

**SUPPLEMENTARY MATERIAL**

**METHODS**

**Data Sources**

Brazilian hematopoietic cell transplant centers report their data to the Center for International Blood and Marrow Transplant Research (CIBMTR) using the electronic FormsNet3 platform. That process is protected by double authentication entry requirements for all system users. The compiled, standardized, and codified data is returned to the Brazilian Society of Cell Therapy and Bone Marrow Transplantation (SBTMO) registry through the Data Back to Centers (DBtC) tool, thereby enabling the analysis of HCT outcomes throughout the country.

**Study Selection**

This retrospective study included all patients diagnosed with MDS who underwent their first allo-HCT in Brazil, from any donor type, between January 1, 2012, and December 31, 2024, as reported for 33 participating centers to the Brazilian Registry of Hematopoietic Cell Transplantation and Cell Therapy Society / Center for International Blood and Marrow Transplant Research (RBTCH-TC/CIBMTR) using the electronic FormsNet3 platform. 238 patients were excluded: 75 who did not undergo a first transplant, 32 with no follow-up data, 123 with no information on post-transplant relapse, and 8 with missing relapse date. Patients who did not undergo a first transplant, had missing follow-up, unavailable post-transplant relapse information, or missing relapse dates were considered exclusion criteria. In total, data from 570 patients were available for analysis. Follow-up was available for 88% of patients at one year and 75% at two years.

Eligibility for HCT was determined independently by each participating center, based on available diagnostic tools and local clinical decision‐making processes. For the purposes of outcome analysis, patients were stratified according to the WHO 2016 classification, chosen given the study’s retrospective design. The MDS/MPN group included patients with overlapping features of myelodysplastic syndromes (MDS) and myeloproliferative neoplasms (MPN), such as chronic myelomonocytic leukemia (CMML), atypical chronic myeloid leukemia (aCML), MDS/MPN with ring sideroblasts and thrombocytosis (MDS/MPN-RS-T), and MDS/MPN unclassifiable (MDS/MPN-U), whereas the MDS group included all other subtypes of myelodysplastic syndromes, such as MDS with excess blasts (MDS-EB), MDS with single lineage dysplasia (MDS-SLD), MDS with multilineage dysplasia (MDS-MLD), MDS with isolated del(5q), and hypoplastic MDS.

**Definitions**

Graft sources were as categorized as bone marrow (BM), mobilized blood cells, or umbilical cord placental blood (UCB). Allogeneic Donors were categorized as grouped into three categories: HLA‐matched related donors, HLA‐mismatched related (including all haploidentical donors and those with a single mismatch at HLA‐A, ‐B, ‐C, or ‐DRB1), and unrelated donors (either matched HLA or mismatched). The classification of the type of conditioning regimen was based on the agents and doses used, as follows: myeloablative conditioning (MAC) for patients receiving total body irradiation (TBI) > 500 cGy in a single exposure or > 800 cGy in fractionated exposures; busulfan > 9 mg/kg orally or ≥ 7.2 mg/kg administered intravenously (IV) or melphalan > 150 mg/m2 IV as a single agent or in combination with other drugs^7^. Reduced‐intensity conditioning (RIC) regimens were defined as TBI < 500 cGy in a single fraction or ≤ 800 cGy in fractionated doses, oral busulfan < 9 mg/kg or IV busulfan < 7.2 mg/kg, melphalan < 140 mg/m2 IV, or thiotepa < 10 mg/kg IV^8^_._ Regimens that did not meet criteria for MAC or RIC were classified as non‐myeloablative (NMA).

**Statistical Analysis**

Descriptive statistics were performed for variables related to patient, disease, and transplantation characteristics. Categorical variables were compared using the Chi-square test or Fisher's Exact Test were conducted. Survival probabilities were estimated by the Kaplan-Meier method and results were compared by the log-rank test with statistical significance considered (p<0.05). The risk factors for mortality included in the analysis were patient age at infusion, gender, donor type, hematopoietic cell transplantation comorbidity index (HCT-CI), graft source, performance status, conditioning regimen type, and disease subtype (MDS vs. MDS/MPN). The univariable analysis was performed by proportional hazards regression (p<0.05), and multivariable analysis was conducted using Cox proportional hazards regression, stepdown method, with selection based on the p < 0.1 threshold, using R Statistical Software (version 4.2.1).

**Ethical Considerations**

All procedures in this study complied with the ethical standards of the institutional and national research committees, in accordance with the revised version of the Helsinki Declaration of 1975. Ethical approval for the use of the CIBMTR platform within the Brazilian Registry for Research was obtained from the National Institutional Review Board (IRB) in 2019 (Conep CAAE: 65575317.5.1001.0071), Principal Investigator Dr. Nelson Hamerschlak).

| **Supplementary Tables** |  |  |  |
| --- | --- | --- | --- |
| **Table S1:** Patient and transplant characteristics of the study cohort (N=570). | | | |
|  | **Diagnosis category** | |  |
|  |  |  |  |
| **Characteristics** | **MDS** | **MDS/MPN** | ***p* value** |
|  | **(n = 476)** | **(n = 94)** |  |
| **Age at transplant, median years (range)** | 50 (23-62) | 59 (45.5-65.8) | **< 0.001*** |
| **Grouped ages, years, n (%)** |  |  | **< 0.001*** |
| -   0 – 17 | 100 (21) | 5 (5.3) |  |
| -   18 – 39 | 68 (14.3) | 11 (11.7) |  |
| -   40 – 59 | 156 (32.8) | 32 (34) |  |
| -   ≥60 | 152 (31.9) | 46 (48.9) |  |
| **Patient sex, n (%)** |  |  | 0.30 |
| -   Female | 215 (45.2) | 37 (39.4) |  |
| -   Male | 261 (54.8) | 57 (60.6) |  |
| **HLA donor type, n (%)** |  |  | 0.243 |
| -   Matched Related | 153 (32.1) | 22 (23.4) |  |
| -   Matched Unrelated | 119 (25) | 27 (28.7) |  |
| -   Mismatched (Haploidentical) | 204 (42.9) | 45 (47.9) |  |
| **Graft cell source, n (%)** |  |  | 0.23 |
| -   Mobilized peripheral blood | 284 (59.7) | 65 (69.1) |  |
| -   Bone marrow | 188 (39.5) | 29 (30.9) |  |
| -   Umbilical cord placental blood | 4 (0.8) | 0 (0) |  |
| **Karnowfsky Performance Status at transplant, n (%)** |  |  | **0.004*** |
| -   100-90 | 383 (80.5) | 65 (69.1) |  |
| -   ≤80 | 86 (18.1) | 23 (24.5) |  |
| -   Missing information | 7 (1.5) | 6 (6.4) |  |
| **HCT-CI at transplant, n (%)** |  |  | 0.269 |
| -   0 | 280 (58.8) | 54 (57.4) |  |
| -   1-2 | 121 (25.4) | 31 (33) |  |
| -   >=3 | 52 (10.9) | 7 (7.4) |  |
| -   Missing information | 23 (4.8) | 2 (2.1) |  |
| **Conditioning regimen type, n (%)** |  |  | 0.052 |
| -   Myeloablative | 271 (56.9) | 47 (50) |  |
| -   Non-myeloablative/reduced intensity | 202 (42.4) | 44 (46.8) |  |
| -   Missing information | 3 (0.6) | 3 (3.2) |  |
| **Survival Status, n (%)** |  |  | 0.244 |
| -   Alive | 284 (59.7) | 50 (53.2) |  |
| -   Dead | 192 (40.3) | 44 (46.8) |  |
| **Note:** p-value Chi-square test, *p<0.05. Abbreviations: HCT-CI, Hematopoietic Cell Transplantation–Comorbidity Index; CMV, Cytomegalovirus; HLA, Human Leukocyte Antigen**.** | | | |

| **Table S2:** Univariate and Multivariate Analyses of Overall Survival (OS), Relapse-Free Survival (RFS), and Non-Relapse Mortality (NRM) in Patients with MDS (n=570). | | | | | | | | | | | |  |
| --- | --- | --- | --- | --- | --- | --- | --- | --- | --- | --- | --- | --- |
| **Disease category** | **OS^a^** | | | | **RFS^a^** | | | | **NRM^a^** | | | |
|  | **Univariate analysis** | | **Multivariate analysis** | | **Univariate analysis** | | **Multivariate analysis** | | **Univariate analysis** | | **Multivariate analysis** | |
|  | **HR (95% CI)** | **p-value** | **HR (95% CI)** | **p-value** | **HR (95% CI)** | **p-value** | **HR (95% CI)** | **p-value** | **HR (95% CI)** | **p-value** | **HR (95% CI)** | **p-value** |
| **Patient.Age.At.Infusion** | 1.01 | 0.000022* |  |  | 1.01 | 0.0000093* |  |  |  |  |  |  |
|  | (1.01 - 1.02) |  |  |  | (1.01-1.02) |  |  |  |  |  |  |  |
| **Age_years[0-17 years]** | 1 |  | 1 |  | 1 |  | 1 |  | 1 |  | 1 |  |
| **Age_years[18-39 years]** | 1.41 | 0.18 | 1.42 | 0.201 | 1.55 | 0.076* | 1.57 | 0.084* | 0.65 | 0.103 | 1.30 | 0.470 |
|  | (0.85 - 2.33) |  | (0.83 - 2.44) |  | (0.96-2.5) |  | (0.94-2.62) |  | (0.39-1.09) |  | (0.63-2.67) |  |
| **Age_years[40-59 years]** | 1.48 | 0.074 | 1.50 | 0.096* | 1.54 | 0.04* | 1.52 | 0.074* | 0.97 | 0.828 | 1.84 | 0.039* |
|  | (0.96 - 2.26) |  | (0.93 - 2.43) |  | (1.02-2.32) |  | (0.96-2.41) |  | (0.7-1.33) |  | (1.03-3.27) |  |
| **Age_years[≥60 years]** | 2.46 | 0.000016* | 2.24 | 0.001* | 2.51 | 0.0000052* | 2.17 | 0.001* | 1.77 | 0.000225* | 2.50 | 0.002* |
|  | (1.64 - 3.71) |  | (1.38 - 3.64) |  | (1.69-3.72) |  | (1.36-3.47) |  | (1.31-2.39) |  | (1.39-4.51) |  |
| **Gender [Female]** | 1 |  |  |  | 1 |  |  |  | 1 |  |  |  |
| **Gender [Male]** | 1.31 | 0.042* |  |  | 1.37 | 0.014* |  |  | 1.32 | 0.083* |  |  |
|  | (1.01 - 1.71) |  |  |  | (1.07-1.76) |  |  |  | (0.96-1.8) |  |  |  |
| **DonorType** | 1 |  | 1 |  | 1 |  | 1 |  | 1 |  | 1 |  |
| **[Matched related]** |  |  |  |  |  |  |  |  |  |  |  |  |
| **DonorType** | 1.05 | 0.75 | 1.22 | 0.245 | 1.03 | 0.87 | 1.20 | 0.252 | 0.96 | 0.827 | 1.32 | 0.140 |
| **[Unrelated]** | (0.77 - 1.43) |  | (0.87 - 1.70) |  | (0.77-1.37) |  | (0.88-1.64) |  | (0.69-1.34) |  | (0.90-1.93) |  |
| **DonorType** | 1.59 | 0.0035* | 1.57 | 0.009* | 1.46 | 0.012* | 1.44 | 0.027* | 1.51 | 0.0147* | 1.66 | 0.013* |
| **[Haploidentical]** | (1.16 - 2.16) |  | (1.12 - 2.21) |  | (1.09-1.96) |  | (1.04-1.99) |  | (1.08-2.11) |  | (1.12-2.47) |  |
| **HCTCI[HCTCI 0]** | 1 |  | 1 |  | 1 |  | 1 |  | 1 |  | 1 |  |
| **HCTCI[T.HCTCI 1-2]** | 1.59 | 0.0017* | 1.26 | 0.147 | 1.5 | 0.0044* | 1.20 | 0.238 | 1.47 | 0.0239* | 1.13 | 0.510 |
|  | (1.19 - 2.13) |  | (0.92 - 1.72) |  | (1.13-1.98) |  | (0.89-1.61) |  | (1.05-2.04) |  | (0.78-1.64) |  |
| **HCTCI[T.HCTCI >=3]** | 1.42 | 0.097* | 1.22 | 0.366 | 1.55 | 0.022* | 1.30 | 0.197 | 1.17 | 0.511 | 1.09 | 0.750 |
|  | (0.94 - 2.14) |  | (0.79 - 1.88) |  | (1.06-2.26) |  | (0.87-1.94) |  | (0.74-1.85) |  | (0.66-1.78) |  |
| **Graft source [Bone marrow]** | 1 |  |  |  | 1 |  |  |  | 1 |  |  |  |
| **Graft source [Mobilized Peripheral blood]** | 1.48 | 0.005* |  |  | 1.46 | 0.0044* |  |  | 1.4 | 0.0421 |  |  |
|  | (1.13 - 1.95) |  |  |  | (1.12-1.89) |  |  |  | (1.01-1.94) |  |  |  |
| **Graft source [Umbilical cord blood]** | 1.81 | 0.41 |  |  | 2 | 0.33 |  |  | 0.98 | 0.986 |  |  |
|  | (0.44 - 7.36) |  |  |  | (0.49-8.12) |  |  |  | (0.1-9.29) |  |  |  |
| **Performance Status[90 ou 100%]** | 1 |  | 1 |  | Referência |  | 1 |  | 1 |  | 1 |  |
| **Performance Status[T.<90%]** | 1.74 | 0.00038* | 1.53 | 0.010* | 1.56 | 0.0031* | 1.35 | 0.063* | 1.81 | 0.00095 | 1.59 | 0.015* |
|  | (1.28 - 2.36) |  | (1.10 - 2.11) |  | (1.16-2.11) |  | (0.98-1.84) |  | (1.27-2.57) |  | (1.09-2.31) |  |
| **Conditioning Type [Myeloablative]** | 1 |  | 1 |  | 1 |  | 1 |  | 1 |  | 1 |  |
| **Conditioning Type [Non-myeloablative/Reduced intensity]** | 1.41 | 0.0091* | 0.99 | 0.948 | 1.48 | 0.0016* | 1.11 | 0.477 | 1.43 | 0.0213* | 1.00 | 1.000 |
|  | (1.09 - 1.83) |  | (0.73 - 1.34) |  | (1.16 - 1.9) |  | (0.83-1.48) |  | (1.05-1.94) |  | (0.70-1.43) |  |
| ***MDS*** | 1 |  | 1 |  | 1 |  | 1 |  | 1 |  |  |  |
| ***MDS/MPN*** | 1.44 | 0,03* | 1.26 | 0,208 | 1.58 | 0,0033* | 1.35 | 0.079* | 1.21 | 0.353 |  |  |
|  | (1.03–2) |  | (0.88–1,79) |  | (1.16–2.14) |  | (0.97–1.88) |  | (0.81–1.79) |  |  |  |
| (a) Cox proportional hazards regression * (p < 0.1).  Abbreviations: HR: Hazard ratio; MDS: myelodysplastic syndromes; MDS/MPN: myelodysplastic/myeloproliferative neoplasms; NRM: non-relapse mortality; OS: overall survival; RFS: relapse-free survival. | | | | | | | | | | | | |
